# Outcomes of Invasive and Noninvasive Ventilation in a Haitian Emergency Department

**DOI:** 10.5334/aogh.4009

**Published:** 2023-10-19

**Authors:** Anna P. Fang, Marie Cassandre Edmond, Regan H. Marsh, Manouchka Normil, Nivedita Poola, Sherley Jean Michel Payant, Pierre Ricot Luc, Natalie Strokes, Manise Calixte, Linda Rimpel, Shada A. Rouhani

**Affiliations:** 1 Boston Medical Center, Department of Emergency Medicine, One Boston Medical Center Place, Boston, MA, USA; 2 Emergency Department, Hôpital Universitaire de Mirebalais, Mirebalais, Haiti; 3 Zanmi Lasante, Port-au-Prince, Haiti; 4 Department of Emergency Medicine, Harvard Medical School, Boston, MA, USA; 5 Partners In Health, Boston, MA, USA; 6 Department of Emergency Medicine, Brigham and Women’s Hospital, Boston, MA, USA; 7 Family Medicine, GHESKIO Centers, Port-au-Prince, Haiti; 8 Department of Emergency Medicine, SUNY Downstate/King’s County Hospital, Brooklyn, NY, USA; 9 Family Medicine, Hôpital Universitaire de Mirebalais, Mirebalais, Haiti

**Keywords:** global health, emergency medicine, respiratory failure, intubation, noninvasive ventilation, Haiti

## Abstract

**Background:**

Limited data exist on the outcomes of patients requiring invasive ventilation or noninvasive positive pressure ventilation (NIPPV) in low-income countries. To our knowledge, no study has investigated this topic in Haiti.

**Objectives:**

We describe the clinical epidemiology, treatment, and outcomes of patients requiring NIPPV or intubation in an emergency department (ED) in rural Haiti.

**Methods:**

This is an observational study utilizing a convenience sample of adult and pediatric patients requiring NIPPV or intubation in the ED at an academic hospital in central Haiti from January 2019–February 2021. Patients were prospectively identified at the time of clinical care. Data on demographics, clinical presentation, management, and ED disposition were extracted from patient charts using a standardized form and analyzed in SAS v9.4. The primary outcome was survival to discharge.

**Findings:**

Of 46 patients, 27 (58.7%) were female, mean age was 31 years, and 14 (30.4%) were pediatric (age <18 years). Common diagnoses were cardiogenic pulmonary edema, pneumonia/pulmonary sepsis, and severe asthma. Twenty-three (50.0%) patients were initially treated with NIPPV, with 4 requiring intubation; a total of 27 (58.7%) patients were intubated. Among those for whom intubation success was documented, first-pass success was 57.7% and overall success was 100% (one record missing data); intubation was associated with few immediate complications. Twenty-two (47.8%) patients died in the ED. Of the 24 patients who survived, 4 were discharged, 19 (intubation: 12; NIPPV: 9) were admitted to the intensive care unit or general ward, and 1 was transferred. Survival to discharge was 34.8% (intubation: 22.2%; NIPPV: 52.2%); 1 patient left against medical advice following admission.

**Conclusions:**

Patients with acute respiratory failure in this Haitian ED were successfully treated with both NIPPV and intubation. While overall survival to discharge remains relatively low, this study supports developing capacity for advanced respiratory interventions in low-resource settings.

## Introduction

Acute respiratory failure is a common presenting condition to the emergency department (ED) and a leading cause of morbidity and mortality in low-income countries (LICs), as recently highlighted by the COVID-19 pandemic [1,2,3,4,5,6]. Effective management of acute respiratory failure in the ED requires an understanding of the underlying causes and the capacity for advanced respiratory support. In high-income countries (HICs), treatment for acute respiratory failure frequently includes noninvasive positive pressure ventilation (NIPPV) or endotracheal intubation [7,8]. NIPPV reduces mortality and the need for intubation among patients able to protect their airway [7]; intubation is definitive management for those who fail or are not NIPPV candidates. Compared to HICs, LICs face significant limitations in providing such advanced respiratory support. NIPPV has been considered as a cost-effective alternative to intubation in such settings and has been investigated in pediatric populations as well as increasingly in middle-income countries [2,6,9,10,11]. However, use of NIPPV or invasive ventilation remains limited in LICs, where EDs often lack the resources to manage patients with advanced respiratory failure, including oxygen, monitors, mechanical ventilators, intensive care units, and trained providers [5,6,12].

To date, few studies have investigated the epidemiology and outcomes of acute respiratory failure managed with NIPPV or invasive ventilation in low- and middle-income countries (LMICs), and the existing data reveal significant variation. Studies published prior to the COVID-19 pandemic demonstrate high burdens of pneumonia, chronic obstructive pulmonary disease (COPD), acute respiratory distress syndrome (ARDS) as well as pediatric sepsis and congenital heart disease [2,13,14]. Reported mortality rates from acute respiratory failure range from 14%–78% [13,14,15]. However, the majority of these studies were conducted in intensive care unit (ICU) settings, and in Haiti, like many LMICs, patients often receive initial and/or ongoing critical care in the ED [12]. The importance of understanding the patterns and outcomes of patients managed with NIPPV or invasive ventilation has been highlighted by the COVID-19 pandemic, where high proportions of patients required advanced respiratory support [6,16].

ED management of acute respiratory failure with noninvasive or invasive ventilation is critical to improving quality of care and patient outcomes. To our knowledge, no study has investigated the etiologies or outcomes of respiratory failure requiring NIPPV or intubation in Haiti. This study aimed to describe the clinical epidemiology, management, and outcomes of patients requiring NIPPV or intubation in the ED at an academic hospital in Haiti.

## Methods

### Study setting

This is a prospective observational study of a convenience sample of patients presenting to the ED at Hôpital Universitaire de Mirebalais (HUM), an academic hospital in central Haiti approximately two hours from Port-au-Prince. HUM serves primary and tertiary catchment areas of 180,000 and two million, respectively, with approximately 14,000 annual visits. At the time of this study, HUM had a 21-bed ED, 5-bed ICU, and was one of few hospitals with ventilatory capacity outside of the operating room. In 2014, HUM launched Haiti’s first Emergency Medicine (EM) residency, a three-year program. The ED is staffed by EM-trained attending physicians, EM residents, and off-service rotating residents. All EM providers are trained in airway management and critical care.

### Patient selection and data collection

Patients requiring NIPPV or intubation in the HUM ED were identified for inclusion between 15 January 2019 and 15 February 2021. Due to significant political unrest in Haiti, then extensive ED renovations, followed by the COVID-19 pandemic, data collection was interrupted February–April 2019, July–September 2019, and March–July 2020. Patients were primarily identified prospectively by ED providers at the time of care. Prospective identification of patients allowed for the study to capture relevant clinical information not routinely recorded in patient charts. Consent was obtained from the patient/patient’s family at the time of care and the provider completed a short questionnaire in real time on clinical information not otherwise documented in the patient chart. All questionnaires were submitted directly into a locked box accessible only to study personnel. In addition to the questionnaire, a research assistant (RA) trained by primary investigators reviewed the charts of all included patients to collect information on triage acuity, diagnosis, comorbidities, clinical management, and outcomes. To reduce the risk of bias from missed patients, the RA also reviewed ICU admission records for eligible patients who were not identified in real time; for these patients, no physician questionnaire was completed and only information in the charts was used for analysis. Patients were followed from arrival to the ED until they left HUM, due to death, discharge, leaving against medical advice, or transfer. Data were extracted into a standardized data collection tool using REDCap [17]. The study was approved by the institutional review boards of Zanmi Lasante in Haiti (Protocol 96) and Mass General Brigham in the United States (Protocol 2018P002471).

### Inclusion and exclusion criteria

This study included any patient with acute respiratory failure treated with NIPPV or intubation in the HUM ED. Those deceased on arrival to the ED were excluded. In March 2020, HUM established a separate COVID-19 unit. Depending on if their COVID-19 diagnosis was known at presentation, some patients were seen initially in the ED and others triaged directly to the COVID-19 unit. Patients with COVID-19 who were intubated in the ED were included in this study; those intubated elsewhere were excluded.

### Patient involvement

Outside of informed consent provided by the patient/patient’s family, patients were not involved in the design or conduct of this study.

### Definitions

Triage acuity was determined by the South African Triage Scale used at HUM; high priority was defined as red or orange [18]. Vitals were analyzed based on normal ranges by age; hypotension was defined as systolic blood pressure <100 for age >12 years, <80 for ages 1–12, and <70 for <1 year. Comorbidities were extracted from patient charts and physician questionnaires into a predetermined list of common conditions.

Presumed diagnoses and indications for NIPPV/intubation were reported by responding physicians. Free-text diagnoses were subsequently classified into cardiovascular, respiratory, infectious, hematological, neurological, endocrine, and other etiologies by research team consensus. Possible indications for respiratory support were predetermined by the study team and providers selected the appropriate indication or wrote an alternate in the questionnaire. For patients managed noninvasively, providers documented patient respiratory status as improved, unchanged, or worsened within 30 minutes of initiating NIPPV. Diagnostic imaging findings were reported by responding physicians and later categorized by the research team based on finding frequency. Difficult airway was defined as obese body habitus, Mallampati class III/IV, spinal immobilization, or assessment via LEMON criteria, which were each reported by the clinician [19]. The LEMON airway assessment is a commonly used airway assessment defined by the following criteria: Look (external evaluation), Evaluate (3-3-2 rule), Mallampati, Obstruction, and Neck mobility [19,20].

NIPPV failure was defined as requiring intubation or death following NIPPV. Survival to discharge was defined as survival to discharge from the ED or hospital, leaving against medical advice, or transfer. Patients are only transferred from HUM if the necessary subspecialty care is unavailable (renal replacement therapy, neurosurgery, or advanced maxillofacial surgery).

### Study outcomes

The primary outcome was survival to discharge. Secondary outcomes for NIPPV included requiring intubation at any time and NIPPV failure rate. Secondary outcomes for intubation included first-pass and overall intubation success rates.

### Data analysis

Data were analyzed in SAS v9.4. Proportions were reported for categorical variables and means and standard deviations for continuous variables. Results were reported for all patients as well as separately for those initially treated with NIPPV and those requiring intubation. Patients receiving both NIPPV and intubation were included in both groups. Chi-squared tests were used to compare survival to discharge based on categorical patient age, gender, prevalent comorbidities, presumed diagnosis, monitoring prior to NIPPV, and NIPPV trial and operator level of training for those intubated. Missing data were excluded. Tests with a *p*-value less than 0.05 were considered statistically significant.

## Results

During the study period, 46 patients treated with NIPPV or intubation were identified (Table 1); 44/46 (95.7%) patients were identified at the time of care and 2/46 (4.3%) via ICU record review. The mean patient age was 31.4 years. Most (69.6%) patients were adult; 14 (30.4%) patients were pediatric (age <18 years). The majority of patients were female (58.7%). Common comorbidities included heart failure, asthma, hypertension, and diabetes. Patients were frequently triaged as high priority. Among those with available data at triage, approximately half had oxygen saturation (SpO2) <90%. Few patients were hypotensive on arrival.

**Table 1:** Patient demographics and triage characteristics.


CHARACTERISTIC	OVERALL	INTUBATION	NIPPV

	N = 46	N = 27 (58.7%)	N = 23 (50.0%)

Age, mean ± SD	31.41 ± 21.42	27.52 ± 20.77	37.43 ± 22.86

Age <1 year, n (%)	3 (6.5%)	3 (11.1%)	0 (0.0%)

Age 1–4 years, n (%)	2 (4.3%)	2 (7.4%)	1 (4.3%)

Age 5–17 years, n (%)	9 (19.6%)	4 (14.8%)	5 (21.7%)

Age 18–24 years, n (%)	4 (8.7%)	2 (7.4%)	2 (8.7%)

Age 25–34 years, n (%)	7 (15.2%)	7 (25.9%)	1 (4.3%)

Age 35–44 years, n (%)	7 (15.2%)	2 (7.4%)	5 (21.7%)

Age 45–54 years, n (%)	9 (19.6%)	5 (18.5%)	5 (21.7%)

Age >55 years, n (%)	5 (10.9%)	2 (7.4%)	4 (17.4%)

Female, n (%)	27 (58.7%)	15 (55.6%)	14 (60.9%)

Comorbidities^a,b^	n = 41	n = 23	n = 22

Any comorbidity, n (%)	21 (51.2%)	9 (39.1%)	13 (59.1%)

Diabetes	3 (7.3%)	3 (13.0%)	0 (0.0%)

Kidney disease	2 (4.9%)	0 (0.0%)	2 (9.1%)

Hypertension	3 (7.3%)	1 (4.3%)	2 (9.1%)

Congestive heart failure	8 (19.5%)	0 (0.0%)	8 (36.4%)

Asthma	4 (9.8%)	3 (13.0%)	2 (9.1%)

Tuberculosis	1 (2.4%)	1 (4.3%)	0 (0.0%)

Other	1 (2.4%)	1 (4.3%)	0 (0.0%)

Number of comorbidities, mean ± SD	0.54 ± 0.55	0.39 ± 0.50	0.64 ± 0.58

SATS triage level^a^, n (%)	n = 41	n = 23	n = 20

Red (highest priority)	30 (73.2%)	17 (73.9%)	15 (75.0%)

Orange	9 (22.0%)	5 (21.7%)	4 (20.0%)

Yellow	2 (4.9%)	1 (4.3%)	1 (5.0%)

Green (lowest priority)	0 (0.0%)	0 (0.0%)	0 (0.0%)

Visit due to trauma, n (%)	0 (0.0%)	0 (0.0%)	0 (0.0%)

Triage vitals			

Systolic blood pressure	n = 33	n = 17	n = 18

mmHg, mean ± SD	133.52 ± 39.65	120.24 ± 26.60	144.78 ± 44.96

Patient hypotensive^c^, n (%)	6 (18.2%)	4 (23.5%)	2 (11.1%)

SpO2	n = 42	n = 24	n = 22

%, mean ± SD	85.12 ± 18.05	84.54 ± 21.70	85.00 ± 13.64

SpO2 <90%, n (%)	20 (47.6%)	10 (41.7%)	12 (54.5%)


SATS, South African Triage Scale; SpO2, oxygen saturation. ^a^ Excludes patients with missing data. ^b^ No patients were reported to have comorbid HIV, epilepsy, or stroke. ^c^ Hypotension was defined as SBP <100 for age >12 years, <80 for ages 1–12, and <70 for <1 year old.

The most common presenting diagnoses were cardiovascular in origin (34.8%), most commonly pulmonary edema (Table 2). Infectious causes including pneumonia/pulmonary sepsis and unspecified bacterial infection accounted for 28.3% of cases, while respiratory diseases including asthma and ARDS (without specified infectious etiology) occurred in 21.7%. Hematological, neurological, and endocrine etiologies were less common. Leading pediatric diagnoses were cardiovascular (35.7%) and infectious (28.6%).

**Table 2:** Presenting diagnosis and indications for advanced respiratory support.


CHARACTERISTIC	OVERALL	INTUBATION	NIPPV

	N = 46	N = 27 (58.7%)	N = 23 (50.0%)

Presumed diagnosis at time of respiratory support^a^, n (%)			

Cardiovascular	16 (34.8%)	4 (14.8%)	13 (56.5%)

Cardiogenic pulmonary edema	12 (26.1%)	1 (3.7%)	11 (47.8%)

Cardiogenic shock	3 (6.5%)	0 (0.0%)	3 (13.0%)

Pulmonary embolism	1 (2.2%)	0 (0.0%)	1 (4.3%)

Hypovolemic shock	3 (6.5%)	3 (11.1%)	1 (4.3%)

Hypertensive emergency	2 (4.3%)	0 (0.0%)	2 (8.7%)

Respiratory	10 (21.7%)	5 (18.5%)	6 (26.1%)

Severe asthma	6 (13.0%)	4 (14.8%)	3 (13.0%)

Acute respiratory distress syndrome	2 (4.3%)	1 (3.7%)	1 (4.3%)

Pulmonary hemorrhage	1 (2.2%)	0 (0.0%)	1 (4.3%)

COPD	0 (0.0%)	0 (0.0%)	0 (0.0%)

Infectious	13 (28.3%)	12 (44.4%)	3 (13.0%)

Pneumonia or pulmonary sepsis	7 (15.2%)	6 (22.2%)	3 (13.0%)

Bacterial infection (unspecified)	2 (4.3%)	2 (7.4%)	0 (0.0%)

Hematological	2 (4.3%)	2 (7.4%)	0 (0.0%)

Neurological	2 (4.3%)	2 (7.4%)	0 (0.0%)

Endocrine	3 (6.5%)	3 (11.1%)	0 (0.0%)

Other	3 (6.5%)	2 (7.4%)	1 (4.3%)

Indication for advanced respiratory support^a^, n (%)			

Hypoxia	–	11 (40.7%)	15 (65.2%)

CO_2_ retention	–	4 (14.8%)	0 (0.0%)

Respiratory distress	–	2 (7.4%)	8 (34.8%)

Respiratory arrest	–	8 (29.6%)	–

Cardiac arrest	–	2 (7.4%)	–

Airway protection	–	12 (44.4%)	–

Airway obstruction	–	3 (11.1%)	–

Other	–	1 (3.7%)	0 (0.0%)


COPD, chronic obstructive pulmonary disease. CO_2_, carbon dioxide. ^a^ Multiple presumed diagnoses and indications for respiratory support could be selected for a patient.

Fifty percent (23/46) of patients were treated with NIPPV and, ultimately, 27/46 (58.7%) were intubated. The most common indications for NIPPV were hypoxia and respiratory distress; common indications for intubation included airway protection, hypoxia, and respiratory arrest (Table 2). Of the 12 patients marked by physicians as intubated for airway protection, 8 had additional indication(s) for advanced respiratory support selected.

Of the 23 patients treated with NIPPV, 22 received BiPAP and 1 CPAP (Table 3). The majority of patients were on SpO2 monitoring prior to NIPPV. Few patients were evaluated with blood gas or chest x-ray prior to treatment, while 65.2% were evaluated with point-of-care ultrasound. Thirty minutes after starting NIPPV, respiratory status was improved in 69.6% patients and unchanged or worsened in 30.4% patients. Four (4/20; 20.0%) patients required intubation. Three of 23 records had incomplete information about whether the patient required intubation following NIPPV.

**Table 3:** Pre-NIPPV monitoring and NIPPV success.


CHARACTERISTIC^a^	NIPPV

	N = 23

Last vitals prior to NIPPV	

Systolic blood pressure, mean ± SD (n = 17)	123.24 ± 61.82

Patient hypotensive^b^, n (%)	4 (23.5%)

SpO2 (%), mean ± SD	88.96 ± 8.90

SpO2 <90%, n (%)	13 (56.5%)

On monitoring prior to NIPPV, n (%)	23 (100.0%)

SpO2 monitoring, n (%)	22 (95.7%)

Blood pressure monitoring, n (%)	15 (65.2%)

Cardiac monitoring, n (%)	4 (17.4%)

Diagnostics and findings prior to NIPPV	

Chest X-ray, n (%)	5 (21.7%)

Bilateral infiltrates (n = 3)	2 (66.7%)

Pulmonary edema (n = 3)	1 (33.3%)

Ultrasound, n (%)	15 (65.2%)

B lines (n = 11)	7 (63.6%)

Pleural effusion (n = 11)	2 (18.2%)

Cardiac finding (n = 11)	2 (18.2%)

Blood gas obtained, n (%)	7 (30.4%)

NIPPV type, n (%)	

CPAP	1 (4.3%)

BiPAP	22 (95.7%)

Patient respiratory status 30 minutes following NIPPV, n (%)	

Improved	16 (69.6%)

Unchanged or worsened	7 (30.4%)

SpO2 <90% prior to NIPPV, n (%)	13 (56.5%)

Achieved SpO2 >90% after NIPPV, n (%)	11 (84.6%)

Patient required intubation, n (%) (n = 20)	4 (20.0%)

NIPPV discontinued during time in ED, n (%) (n = 19)	2 (10.5%)


SpO2, oxygen saturation; CPAP, continuous positive airway pressure; BiPAP, bi-level positive airway pressure. ^a^ For characteristics with missing information, the number of patients with available information is noted in parentheses. ^b^ Hypotension was defined as SBP <100 for age >12 years, <80 for ages 1–12, and <70 for <1 year old.

Intubation characteristics are shown in Table 4. The majority of patients were preoxygenated prior to intubation. A formal difficult airway assessment was completed in 48.1%; 37.0% of all patients had a difficult airway anticipated. Initial intubation attempts were frequently completed by second-year EM residents and less frequently by third-year residents. Induction and paralytic agents were used in 19/22 (86.4%) and 15/22 (68.2%) of intubations, respectively. Commonly used drugs are listed in Table 4.

**Table 4:** Pre-intubation management and intubation characteristics and complications.


CHARACTERISTIC^a^	INTUBATION

	N = 27

Preoxygenation before intubation, n (%) (n = 26)	26 (100.0%)

Nasal cannula	2 (7.7%)

Non-rebreather mask	4 (15.4%)

Simple face mask	4 (15.4%)

BiPAP	2 (7.7%)

Bag valve mask, passive	2 (7.7%)

Bag valve mask, active	9 (34.6%)

Other	3 (11.5%)

Last vitals prior to intubation	

Systolic blood pressure, mean ± SD (n = 17)	132.06 ± 40.70

Patient hypotensive^b^, n (%)	3 (17.6%)

SpO2 (%), mean ± SD (n = 22)	94.50 ± 7.95

SpO2 <90%, n (%)	3 (13.6%)

Blood gas obtained, n (%)	10 (37.0%)

pH, mean ± SD (n = 7)	6.99 ± 0.49

CO_2_, mean ± SD (n = 7)	52.73 ± 23.68

Formal difficult airway assessment completed, n (%)	13 (48.1%)

Difficult airway anticipated^c,d^, n (%)	10 (37.0%)

Number of risk factors for difficult airway^e^, mean ± SD	0.89 ± 1.60

Operator on initial attempt	

EM intern	1 (3.7%)

EM second-year resident	16 (59.3%)

EM third-year resident	10 (37.0%)

EM-trained attending	0 (0.0%)

Operator on successful attempt (if 1st attempt unsuccessful)	

Same as initial attempt	2 (7.4%)

EM intern or second-year resident	1 (3.7%)

EM third-year resident	6 (22.2%)

EM-trained attending	2 (7.4%)

First-pass success, n (%) (n = 26)	15 (57.7%)

Among second-year residents	9 (60.0%)

Among third-year residents	6 (60.0%)

Total number of attempts, mean ± SD	1.75 ± 0.94

Overall intubation success, n (%) (n = 26)	26 (100.0%)

Any induction agent administered^f^, n (%) (n = 22)	19 (86.4%)

Ketamine	16 (72.7%)

Midazolam	2 (9.1%)

Propofol	1 (4.5%)

Fentanyl	1 (4.5%)

Any paralytic agent administered^g^, n (%) (n = 22)	15 (68.2%)

Succinylcholine	10 (45.5%)

Vecuronium	5 (22.7%)

Both induction and paralytic agents administered, n (%) (n = 21)	15 (71.4%)

Lowest O2 saturation during intubation (%), mean ± SD (n = 23)	93.61 ± 7.33

Lowest O2 saturation <90%, n (%)	4 (17.4%)

Any method of intubation confirmation^h^, n (%) (n = 21)	21 (100.0%)

Auscultation	21 (100.0%)

Tube condensation	13 (61.9%)

Radiography	2 (9.5%)

CO_2_ detector	0 (0.0%)

Any complication^i^, n (%) (n = 26)	2 (7.7%)

Bleeding	1 (3.8%)

Hypoxia (SpO2<90%)	1 (3.8%)


BiPAP, bi-level positive pressure ventilation; SpO2, oxygen saturation; CO_2_, carbon dioxide. ^a^ For characteristics with missing information, the number of patients with available information is noted in parentheses. ^b^ Hypotension was defined as SBP <100 for age >12 years, <80 for ages 1–12, and <70 for <1 year old. ^c^ Difficult airway was defined as an abnormal LEMON assessment, if LEMON assessment was completed; if assessment was not completed, difficult airway included Mallampati Class III/IV; spinal immobilization; or obese body habitus. ^d^ The LEMON method is an airway assessment defined by the following criteria: Look, Evaluate, Mallampati, Obesity or obstruction, and Neck mobility. ^e^ Risk factors included external evaluation, 3-3-2, Mallampati score (3 or 4), obesity, and neck immobility/spinal immobilization. ^f^ No providers reported using lorazepam, etomidate, or morphine for induction. ^g^ Rocuronium is not available for use at HUM. ^h^ Multiple methods of confirmation could be selected. ^i^ No providers reported complications of direct airway injury, cardiac arrest, cricothyroidotomy, dental trauma, hypotension, or laryngospasm.

The overall intubation first-pass success rate was 57.7% (15/26) and the overall success rate was 100.0% (26/26); 1 record was missing information on intubation outcome (Table 4). Mean number of attempts was 1.75 and maximum was 4. Immediate post-intubation complications occurred in 2/26 intubations, with 1 report of bleeding and hypoxia each (one record missing information).

**Figure 1:**
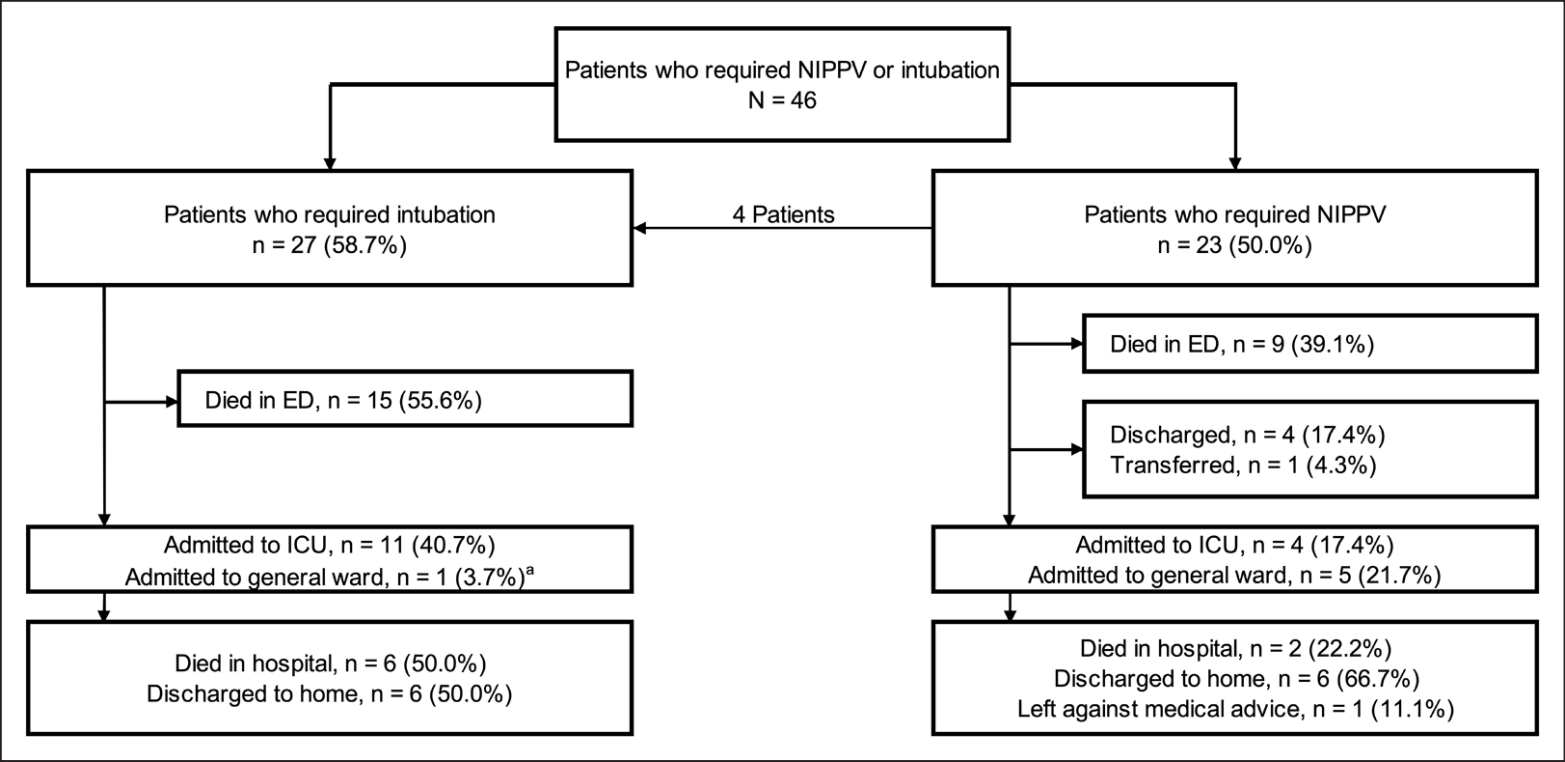
Flowchart of patients presenting with respiratory failure to the HUM ED. ^a^ Patient remained in the ED for 3 days prior to stabilization and transfer to the general ward.

Of 46 patients with acute respiratory failure requiring NIPPV or intubation, a total of 22 (47.8%) died in the ED: 15/27 (55.6%) patients who were intubated and 9/23 (39.1%) patients initially treated with NIPPV (Figure 1). Of the 24 patients who survived, 13/24 (54.2%) were admitted to the ICU and 6/24 (25.0%) to the general ward, after improvement with ED treatment. Of those admitted, 8/19 patients (42.1%) died, 10/19 (52.6%) were discharged to home, and 1 (5.3%) left against medical advice. Thirteen patients (13/23, 56.5%) failed NIPPV (required intubation or died). Of those who failed NIPPV, 9 (69.2%) were marked by responding physicians as hypoxic respiratory failure.

The overall rate of survival to discharge was 34.8% (16/46): 22.2% (6/27) in the intubation group and 52.2% (12/23) in the NIPPV group. Survival to discharge rates were 37.5% (12/32) in adults and 28.6% (4/14) in pediatric patients. The average age of survivors was 33.0 ± 21.0 years. Table 5 compares survival to discharge for the NIPPV and intubation groups among age groups, gender, prevalent comorbidities, triage acuity, presumed etiology, monitoring prior to NIPPV, trial of NIPPV prior to intubation, and intubation operator level of training. Among those requiring NIPPV, patients with comorbid congestive heart failure (*p* = 0.035) and cardiovascular etiologies (*p* = 0.019) were less likely to survive to discharge. Among those requiring intubation, patients with comorbid asthma (*p* = 0.002), respiratory etiologies (*p* < 0.001), and those trialed on NIPPV prior to intubation (*p* = 0.010) were more likely to survive.

**Table 5:** Bivariate analysis of factors associated with survival to discharge.


	NIPPV				INTUBATION			
	
	n	SURVIVED TO DISCHARGE	DID NOT SURVIVE TO DISCHARGE	*P* VALUE^a^	n	SURVIVED TO DISCHARGE	DID NOT SURVIVE TO DISCHARGE	*P* VALUE^a^
	
		N = 12	N = 11			N = 6	N = 21	

Age								

Age <5 years	n = 1	1 (100.0%)	0 (0.0%)	0.328	n = 5	1 (20.0%)	4 (80.0%)	0.895

Age 5–18 years	n = 5	2 (40.0%)	3 (60.0%)	0.538	n = 4	1 (25.0%)	3 (75.0%)	0.885

Age >18 years	n = 17	9 (52.9%)	8 (47.1%)	0.901	n = 18	4 (22.2%)	14 (77.8%)	1.000

Female gender	n = 14	6 (42.9%)	8 (57.1%)	0.265	n = 15	5 (33.3%)	10 (66.7%)	0.121

Comorbidities								

Congestive heart failure	n = 8	2 (25.0%)	6 (75.0%)	0.035	–	–	–	–

Asthma	n = 2	2 (100.0%)	0 (0.0%)	0.176	n = 3	3 (100.0%)	0 (0.0%)	0.002

High priority triage acuity^b^	n = 19	10 (52.6%)	9 (47.4%)	0.305	n = 22	4 (18.2%)	18 (81.8%)	0.639

Presumed diagnosis								

Cardiovascular	n = 13	4 (30.8%)	9 (69.2%)	0.019	n = 4	1 (25.0%)	3 (75.0%)	0.885

Respiratory	n = 6	5 (83.3%)	1 (16.7%)	0.076	n = 5	4 (80.0%)	1 (20.0%)	<0.001

Infectious	n = 3	2 (66.7%)	1 (33.3%)	0.590	n = 12	1 (8.3%)	11 (91.7%)	0.121

On SpO2 monitoring prior to NIPPV	n = 22	12 (54.5%)	10 (45.5%)	0.286	–	–	–	–

Trial of NIPPV before intubation	–	–	–	–	n = 7	4 (57.1%)	3 (42.9%)	0.010

Initial operator first- or second-year resident	–	–	–	–	n = 17	3 (17.6%)	14 (82.4%)	0.456


SpO2, oxygen saturation. ^a^ *P*-values were calculated using chi-squared tests. ^b^ High priority triage acuity includes red and orange triage levels. Excludes patients who had missing data on South African Triage Scale triage level.

## Discussion

This is the first study to evaluate the epidemiology and outcomes of acute respiratory failure requiring NIPPV or intubation in Haiti. It demonstrates that cardiogenic pulmonary edema, pneumonia/pulmonary sepsis, and asthma are common causes of presentations with respiratory failure requiring advanced respiratory support in the ED. Acute respiratory failure treated with NIPPV or intubation was associated with high mortality; however, approximately half of those who survived to hospital admission were ultimately discharged home. This study supports the importance of capacity for airway management by trained ED providers in LICs, given burden of disease, as well as demonstrated high success and low immediate post-intubation complication rates.

The leading cause of respiratory failure in this study was cardiovascular, largely due to cardiogenic pulmonary edema. This is higher than observed in existing studies in LICs; in a study of acute hypoxemic respiratory failure at a tertiary hospital in Uganda, 8.5% presented with cardiac disease [13]. This likely reflects the high burden of heart disease and advanced heart failure in Haiti [3]. Similar to other studies, our study also showed a high burden of infection-related respiratory failure, with frequent diagnoses of pneumonia or sepsis. Infection was also a leading diagnosis in the pediatric population, consistent with the known burden of respiratory infection-related emergencies in pediatric populations [2,13,21,22].

The low survival to discharge rate in this study mirrors other studies. In the aforementioned study in Uganda, acute respiratory failure was associated with a 22.3% survival to discharge rate and a 85% 90-day mortality rate [13]. A study of pediatric (age <18 years) respiratory compromise in an urban ED in Tanzania in which 14.7% of patients required intubation reported a 30.9% hospital mortality rate [14]. Of the 30 patient deaths observed in this study, the majority (22/30) occurred in the ED. From our experience, this is due to limited ICU space to accept unstable patients, leading sick patients to stay in the ED longer, as well as the high acuity and often poor prognosis of patients on arrival. Furthermore, many patients presented with severe heart failure, for which HUM has limited therapeutic options, as treatments for advanced heart failure in HICs such as inotropic medications, devices/pacemakers, and heart transplant are not available. While the overall survival rate is low compared to HICs, the results of this study support the development of ventilatory capacity in LICs [23]. Survivors to discharge were relatively young with a mean age of 33 years, representing many life-years saved.

In this study, 50% of patients were trialed on NIPPV. These patients reflect the recommendations for NIPPV in acute respiratory failure, with appropriate use in populations proven to benefit such as those with pulmonary edema [7,8]. A small number of patients with pneumonia were also trialed on NIPPV despite evidence suggesting high risk of NIPPV failure with pneumonia [7]. This likely reflects the limited resources in this setting and the need to reserve intubation for patients unable to protect their airway.

The NIPPV failure rate reported in this study is higher than reported in existing studies. In a large meta-analysis of NIPPV in LMICs, the pooled overall risks of NIPPV failure and intubation were 28.5% and 28.8%, respectively [2]. Notably the reported risk of failure was much greater in hypoxemic (42.1%) vs. hypercapnic respiratory failure (20.2%), which is consistent with the high prevalence of cases in this study where providers indicated hypoxia as the indication for advanced respiratory support and the high failure rate observed in this study. ED mortality rates with NIPPV in this study were also higher than those reported in the meta-analysis [2]. These discrepancies are likely explained by differences in setting and acuity; the meta-analysis included studies on NIPPV used in the peri-extubation period and the majority were conducted in urban academic ICUs from middle-income countries. Additionally, while HUM is one of few hospitals in Haiti with the capacity to manage intubated patients, the number of available ventilators remains extremely small. As a result, patients with irreversible conditions are not considered candidates for intubation and instead receive maximal medical management and NIPPV. Unfortunately, many do not survive. Low intubation rates have been similarly noted in LICs, where lack of resources often forces early triage decisions regarding patients’ likelihood of survival [24].

Indications for intubations in this study were similar to other studies [25], although fewer intubations occurred in the setting of trauma, cardiac arrest, or neurological injury than reported in other LICs [26,27,28]. This likely correlates with the extremely limited neurosurgical care, making intubation for head trauma uncommon, as well as the limited number of overall available ventilators. However, it suggests an area for future analyses and care investments given the high burden of traumatic injuries in Haiti [29].

The overall intubation success rate (100%) was consistent with existing literature; [25,27] the first-pass success rate (57.7%) was lower than in other LICs. Studies from South Africa, Nigeria, Thailand, Pakistan, and Malaysia reported first-pass success rates ranging from 79.5%–94% [25,26,27,28,30]. Our lower first-pass success rate may be due to higher numbers of trainees completing intubations; intubations in the existing literature were frequently completed by anesthesiologists, senior residents, or attending physicians. Among existing data on trainees, first-pass success rates in this study were also lower; at an academic Malaysian ED, overall first-pass success among EM trainees was 80.6%, although the level of trainees included is unclear [26]. Differences may also be due to a higher percentage of patients with difficult airways or a lower paralytic use rate in this study [25,31]. Although we did not collect data on why paralytics were not used, informal discussions with staff suggest HUM’s lower rates of paralytic use may be due to frequent crash intubations, including during codes.

Immediate post-intubation complication rates were lower compared to existing literature, where reported rates range widely from 5.3%–93.6% [25,26,28,30]. In an international meta-analysis of ED intubations, the most commonly reported peri-intubation complications were hypoxia, hypotension, and esophageal intubation [32]. We report only two immediate complications, hypoxia and bleeding. Low complication rates could be due to extensive training including simulation on airway management for HUM EM residents or due to the small sample size.

This study’s results should be considered relative to its sample size and design. Our sample size was below our target and therefore underpowered to detect predictors of survival. This was likely due to significant pauses in data collection and associated decreased patient volumes due to political unrest and the COVID-19 pandemic. Furthermore, this study only included those treated with NIPPV or intubation; patients with acute respiratory failure who were not candidates for intervention and/or if equipment was unavailable were excluded. This study relied on providers to identify eligible patients as a convenience sample; given the significant ED workload, it is possible that not all patients were identified. We reduced this bias by including the retrospective review of ICU data for eligible patients not identified by providers at the time of care. As only two additional patients were identified in this way, we believe that the risk of selection bias is low, but unknown selection bias may still exist as a result of the convenience sample methodology. Additionally, patients with known or highly suspected COVID-19 were triaged to the COVID-19 unit and therefore their management and outcomes are excluded from this study.

Although providers completed one data collection form in real time, we relied on chart review to capture additional clinical details. As a result, this study was highly dependent on the quality of patient charts. This study provided formal RA training and used a standardized data extraction form for chart information. Use of the questionnaire to report clinical changes and complications may have been subject to observation and recall biases. This was minimized by having providers complete and deposit forms in real time directly into a locked box. Despite these limitations, these results provide valuable initial insight into the management and outcomes of acute respiratory failure in Haiti.

This is the first study to evaluate the etiologies, management, and outcomes of acute respiratory failure treated with noninvasive or invasive ventilation in Haiti. Although this study reports low rates of survival to discharge, the high intubation success rate and young age of survivors support the use of advanced respiratory support in LICs. Larger studies are needed to determine the prevalence of acute respiratory failure and factors associated with survival in Haiti. Immediate future interventions should focus on educational efforts targeting management of common etiologies of acute respiratory failure. Given the demonstrated feasibility of ventilation in this study, future studies based in implementation science are warranted to identify strategies to integrate ventilation for acute respiratory failure across Haiti and in other low-resource settings.
